# Adenoid Vegetations of the Naso-Pharynx1Read to the Buffalo Medical and Surgical Association, February 11, 1890.

**Published:** 1890-04

**Authors:** W. Scott Renner

**Affiliations:** 361 Pearl Street; Buffalo, N. Y.


### ADENOID VEGETATIONS OF THE NASO-PHARYNX.[1]

By W. SCOTT RENNER, M. D., C. M., Buffalo, N. Y.

These growths are simply hypertrophy of the lymphoid tissue found normally abundant during childhood in the vault of the pharynx. They occur in two forms. The first of these is merely hypertrophy of the pharyngeal or Luschka's tonsil, and presents, on examination, a broad sessile outgrowth from the Basilar process of the occipital bone, which may vary in size from a slight enlargement of

1. Read to the Buffalo Medical and Surgical Association, February 11, 1890.

the tonsil to a mass filling the naso-pharynx. At times furrows may be seen on the surface of this tumor, extending antero posteriorly. The middle one of these often ends in a depression at its posterior extremity, from which mucus, or muco-pus, may be seen discharging. The second form occurs as a mass of more or less pedunculated outgrowths from a lymphoid base. These are attached to the roof, posterior and side walls of the naso-pharynx, and, according to some authors, to the posterior margin of the septum narium. They represent not only hypertrophy of the pharyngeal tonsil, but of all the lymphoid tissue of the vault.

The clinical manifestations of these growths and their association with other and similar conditions are such, that it becomes necessary to refer to the distribution of the lymphoid tissue in the throat to appreciate the history of many of the more interesting cases.

Luschka's tonsil is the upper part of the ring of lymphoid tissue described by Waldeyer and Bickel, which extends along the roof and lateral walls of the pharynx from the Basilar process to the base of the tongue, and includes the masses of lymphoid tissue known as the pharyngeal tonsil, the tubal tonsils of Gerlach, the faucial tonsils, and the lymphoid masses, improperly called the lingual tonsils. These are connected together by a net-work of lymphatic vessels and lymphoid follicles; from this ring lymphoid tissue extends to the floor of the mouth and into the nose, in some cases as far as the epiglottis and false cords, and to the anterior extremities of the lower and middle turbinated bodies. The lymphoid tissue of the velum palati forms, with the lower part of this ring, an inner and smaller ring.

The bursa pharyngea was first described by Luschka, as a pouch imbedded in the pharyngeal tonsil and connected by a band of connective tissue with the basilar process of the occipital bone. Ganghofner denied the existence of the bursa as described by Luschka, and considered it a more or less well-marked depression of the mucous membrane covering the pharyngeal tonsil, having no great depth and no connection with the basilar process by means of a band of connective tissue. In 1885, Thornwald published a monograph in which he stated that of 892 cases of naso-pharyngeal disease, he had treated 157 for hypersecretion and forty-five for cystic formation of the bursa pharyngea. This statement engendered much careful investigation as to the constancy of this condition, anatomically on the cadaver, fetus and embryo; clinically more careful examinations have been made with the rhinoscopic mirror. Schwabeck, prominent among the anatomists, has demonstrated the configuration of the tonsil described by Wendt and Ganghofner, *i. e.*, a surface marked by numerous furrows and ridges of various depths, these gradually disappear as age advances, and that the bursa of Thornwald, when present, is but a remnant of the middle pharyngeal cleft of embryonic life, being its posterior end, formed by the partial union of its margins, and not a special anatomical formation; into this depression the fissures of the tonsil, especially the middle one, extend and give it, when the tonsil is diseased, the appearance of a discharging bursa. Clinicians,—Schafer, of Bremen, and many others,—have also failed to demonstrate its constancy, and likewise regard its presence as a part of the disease of the tonsil or a congenital defect.

I have felt it necessary to refer to the anatomy of this part of the throat in order that the special objects of this paper might be more easily understood. I wish to direct your attention to the importance, clinically, of adenoid vegetations and of the condition of the bursa pharyngea, and of the faucial and lingual tonsils in some of the cases of which I shall speak.

As clinicians, our duty is to seek the cause of complaint, remove it if possible, so that the symptoms and sequelæ may disappear, or be modified, if of so long standing that they have produced permanent structural changes. I shall follow this order in discussing these growths, *i. e.*, I shall speak, first, of the signs, etc., which call for a special examination of this part of the pharynx. Secondly, of the method of treatment, and finally of the symptoms and sequelæ individually with the effects of the prescribed treatment upon them.

The symptoms of this condition divide themselves naturally into direct and indirect. The direct include catarrhal discharge from the naso-pharynx, and nasal obstruction, *i. e.*, obstruction of respiration, phonation, olfaction and obstruction of the Eustachian tubes; these express themselves, clinically, as mouth-breathing, defective vocal resonance, loss or partial loss of the sense of smell, and catarrh of the Eustachian tubes and middle ear, with their consequences, the indirect and reflex symptoms of adenoid vegetations. Nasal obstruction is caused by all nasal and naso-pharyngeal growths, whether hypertrophic, neoplastic, or congenital; therefore it cannot be of much value in suspecting the nature of the growth before a physical examination is made, unless accompanied by the "dead toneless voice" and the facial expression which has been described as pathognomonic of this condition, neither of which, however, are well marked unless a very large amount of adenoid growths, or an associated hypertrophy of the faucial tonsils be present. What, then, should make one suspect adenoids in a case of nasal obstruction? First, the age of the patient. They occur usually in children between five and fifteen years of age, sometimes as early as the fourth year, quite frequently between the fifteenth and twentieth year, and where resolution is delayed between the twentieth and the twenty-fifth year. Cases beyond twenty-five are the exception. Since I came to Buffalo, less than four years ago, 110 patients have consulted me for this condition. Of these, seventy-four were under fifteen years of age, twenty-two between fifteen and twenty; thirteen over twenty and under twenty-five years of age; one was forty years of age. The oldest patient on whom I have operated was a young man in his twenty-fourth year. I saw Schroetter operate on an actress of thirty. It has been found necessary to operate on cases in their forty-fifth year. In the case forty years of age the patient was under treatment for a deviation of the septum and hypertrophic rhinitis. Some enlargement of the pharyngeal tonsil was meanwhile discovered, with a copious discharge from its surface. In this case I cauterized the tonsil, not to relieve obstruction but to destroy the source of naso-pharyngeal catarrh. Cohen, of Philadelphia, mentions a case which he had under treatment for goitre. This was in a lady of seventy. On examining the naso-pharynx, he found some enlargement of the pharyngeal tonsil.

Of 500 cases of diseases of the throat and nose treated by me at the Buffalo Eye and Ear Infirmary, 126 were children under fifteen years of age, of whom seventy-six complained of nasal obstruction; this was due in forty-nine cases to adenoids, simple or complicated; in eight cases to simple hypertrophic rhinitis, in children twelve, thirteen and fourteen years of age; in one case to traumatic deviation of the septum; in the remaining cases to specific inflammation of the nose in young children. Of the patients between fifteen and twenty years old, twenty-five per cent. had adenoids; of those between twenty and twenty-five, nine per cent. had adenoids in sufficient quantities to require operative interference.

It is, therefore, evident from my experience, as well as from the evidence of others, that we should expect to find adenoid growths where children complain of nasal obstruction. First, because the lymphoid tissue usually hypertrophies during childhood, and unless there be some reason for delayed resolution, atrophies between puberty and the twentieth year; secondly, because other common forms of nasal obstruction, hypertrophic rhinitis and nasal polypi, are rare before the fifteenth year; and when nasal obstruction occurs during childhood from other causes than these growths, it is usually due to some congenital malformation or constitutional taint producing specific inflammation.

Another, the second reason which should suggest adenoid vegetations, especially in children, is the presence of hypertrophied faucial tonsils. Of thirty-nine cases of enlarged tonsils in children under fifteen, thirty-one or about eighty per cent. had sufficient amounts of adenoid growths to require operation; or, of the 110 cases of adenoids thirty-five had marked hypertrophy of the faucial tonsils. The reasons for suspecting the association of these two are evident: both generally occur before puberty, the resolution of both usually takes place at or soon after puberty, they are similar in structure and probably in function, and causes productive of hypertrophy of one are active in producing a similar condition of the other, as diphtheria, scarlet fever, whooping cough, etc., and probably climatic influences, bad hygienic surroundings, and heredity. The majority of my cases have been ill-nourished and poorly-housed infirmary patients.

A third condition, when found, which should suggest the presence of these growths is adenoid thickening of the fauces. This may occur in various degrees, from a marked thickening of the posterior and side walls of the pharynx to but a few scattered granular follicles. Marked thickening of this nature I have generally found with the pedunculated form of the growths. It indicates merely that there is a general hypertrophy of the lymphoid tissue of the throat.

When led by the above conditions to suspect adenoids in children, the completion of our diagnosis is not difficult; when a good view of the naso-pharynx can be obtained with the rhinoscopic mirror, a papillomatous-looking tumor may be seen obstructing our view of a part or the whole of the picture of the naso-pharynx and posterior nares. Failing to introduce the mirror, the diagnosis may be easily and quickly completed by introducing the left index finger into the naso-pharynx; a practised finger will easily recognize the growth, its consistency and amount, as well as its relation to the Eustachian tubes and posterior nares. In children we are not likely to meet any other condition; after puberty we may rarely meet, in the naso-pharnyx, with some one of the solid tumors; these occur between the fifteenth and twenty-fifth year. Fibroma, the most common of these, is an extremely rare growth, and is easily recognized by its dark red color and smooth surface. I have met but one case of true fibroma of the naso-pharynx in Buffalo. Having diagnosticated the presence of these growths, they should be removed surgically. Various topical applications of iodine and caustics are advised. These have given me so little satisfaction, that I make it a rule to operate or not to treat the case. I have operated on seventy-three, less than two-thirds of my cases. Many more are pending operation, many refused to undergo operation and have been lost sight of.

I do not usually institute any preparatory treatment before operating; tonics will not improve the general health, cough mixtures and sprays will not relieve the catarrhal symptoms until the source of trouble is radically removed, then the patients will usually soon learn to breathe naturally and will regain their general health without any internal medication whatever, provided they have plenty of fresh air, good food, and hygienic surroundings. Various instruments have been advised, but success in operating seems to depend more upon the manipulative skill of the operator, than upon the form of instrument used. I shall confine my remarks to the method of operating which I follow. When possible, I operate with the patient sitting upright, after having applied cocaine to the naso-pharynx (some patients prefer the slight pain of the operation to the disagreeable sensation produced by cocaine); when it is impossible to operate in this way, on account of the age or fear of the patient, I operate with the patient under complete chloroform anesthesia (when the faucial tonsils are enlarged, they should, in either case, be removed a few days previously).

When no anesthetic is required, I use Chiaris' very simple snare with the loop at the end of the curved canula bent toward me, introducing the end of the canula along the back wall of the pharynx into the naso-pharynx until I see the loop encircle the growth, or a portion of it, and complete the operation; this, when necessary, may be repeated at the same sitting, or deferred for a day or so. The patient is able to assist me in obtaining a view of the parts by depressing the tongue while I hold the mirror in position. Where the inferior meatus is spacious, I often introduce the straight canula of the same instrument through the nose, the patient assisting me in the same manner as when operating with the bent canula, by holding down the tongue while I introduce the rhinoscopic mirror into the pharynx. In either of these ways the operation is usually completed in from one to four sittings. Sometimes I use Catti's forceps instead of the snare, and in the same manner. These forceps I use universally when the patient is anesthetised. As soon as the patient is anesthetised, my assistant draws the patient's head well over the elevated end of the operating table or a bolster, introduces Marchoni's gag, which I prefer, on account of its simplicity, (it is easily held and quickly removed with one hand by the assistant) while he manages the head of the patient with the other. As soon as the patient is in this position, I proceed to operate with the forceps, keeping the pharynx lighted by means of a head-mirror to prevent my grasping the uvula and to observe the amount of hemorrhage. After having removed all that I can safely with the forceps, I introduce my index finger and tear away any of the tissue which I may find remaining. I prefer the finger to the curette, on account of the great advantage afforded by the tactile sense. As soon as there is any hemorrhage of importance, or when the operation is completed, the gag is quickly removed and the patient turned on the side so that the blood may run from the mouth and nose, in doing which care must be taken not to compress the chest.

The after-treatment consists of an alkaline spray. I am not in the habit of putting the patients in bed after the operation, as advised by some. Slight fever may follow, but the hemorrhage is usually trivial at the time of the operation and does not return. In one of my cases, a girl of seventeen, hemorrhage set in a couple of hours after the operation, and might have been serious but for the kindness of a neighboring physician, who had plugged the posterior nares before I reached the patient. In this case the growth was unusually dense and bled quite freely at the time of the operation.

The symptoms due to hypersecretion disappear quickly after the operation is completed and the wound healed. These manifest themselves as a discharge of tenacious mucus, or muco-pus, into the pharynx, or as an accumulation of mucus, often dried and inspissated in the nose. The presence of these produce reflex efforts to clear the throat and nose. The patient cannot clear the nose by blowing it. Cough, and especially that form known as "nocturnal nasal cough," is produced by the dropping of mucus into the pharynx at night, long after the patient has fallen asleep. Such a cough must not be mistaken for that due to elongated uvula, hypertrophy of the faucial tonsils, reflex ear cough, or cough due to enlargement of the lingual tonsils, a condition more common in middle-aged women, but rare in children. I have met it but five times in children. In only two of these cases it produced symptoms. One of these, a case in point, I shall relate:

An extremely nervous girl of fourteen was referred to me by Dr. Heath. She was slightly hoarse, but her chief complaint was that, when she had but a slight cold she was troubled with a continuous "dry hacking cough" as soon as she laid down. This persisted as long as she remained lying; a tendency to cough was always present at other times, and she was always making efforts to relieve herself of some irritation in the throat, which she could not describe. When two years of age she had whooping cough, and had been troubled with this cough more or less ever since, and was often compelled to sleep tied in a chair during the whole night. On examining her throat I found some adenoid growths, moderate enlargement of the faucial tonsils, marked enlargement of the lingual lymphoid nodules, and moderate increase in the size of the thyroid gland. I concluded that the cough was probably due to pressure of the nodules at the base of the tongue against the epiglottis when the patient laid down. I determined to confirm the truth of my suspicion by first treating only the lingual tonsil. As soon as this was perceptibly diminished in size by cauterization, the cough had completely disappeared, and that within a week. The adenoids were subsequently removed. The patient has since improved greatly in general health, is much less nervous, much of the enlargement of the thyroid has disappeared, her voice is perfectly clear, she complains no longer of the sensation of a foreign body in the throat, and has not coughed since the first week of treatment. Furthermore, she has gained twenty pounds in weight, without any internal medication whatever.

I mentioned when speaking of the sessile form of growth that a crypt-like depression was often seen in the center, discharging mucus, etc. This I have been able to differentiate in several cases. Should the sides of the ridges of the tonsil become adherent from any cause, might not a retension cyst be the result? As an illustration of which I shall relate the only case of cyst in this region which has come under my observation. This was that of a young lady, nineteen years of age. She was very anemic, had had otitis media purulenta for several years, and complained of nasal obstruction, and the "dead, toneless voice" of adenoids. On examination, a broad sessile growth of pale color was found in the naso-pharynx, which I concluded to be adenoid vegetations, and proceeded to operate. At the first sitting I removed, with the adenoid tissue, a mass of gummy matter about the size of a medium-sized cherry, which I found, on microscopic examination, to consist chiefly of cholesterine crystals and cells in various stages of fatty degeneration. After the operation was completed she improved rapidly in general health. The otitis has not returned, and her voice, although not as clear as it should be, is rapidly regaining its resonance. The mass found in this tonsil resembled that found in obstructed sebaceous glands and in the crypts of faucial tonsils in chronic follicular tonsillitis. This case was probably of the same nature as chronic follicular tonsillitis, in which the secretion was retained by the adhesion of the margins of the middle furrow of the enlarged pharyngeal tonsil, and not a cystic distension of any bursa of this region.

The effects of an operation on mouth-breathing are often immediate; natural respiration is often restored within twenty-four hours. I have seen great improvement after even a small portion of the growth has been removed. There are many cases, however, where it is necessary to constantly remind the patients how they should breathe. Its persistence is often due to a paresis of the dilators of the alæ nasi from want of use.

Mouth-breathing, as a symptom of adenoids, or any condition causing nasal obstruction, produces irritation and congestion of the mucous membrane of the respiratory apparatus from the pharynx to the lungs, on account of the patient's breathing air of improper temperature, moisture and purity; it lessens the force of respiration, shortens and increases the number of inspirations and expirations, thus producing fatigue of the muscles of respiration and speech; but, on account of the age of most patients, if the amount of growth be excessive or associated with hypertrophy of the faucial tonsils, it produces additional effects upon the developing child. The chest may be compressed, narrow or "pigeon breasted," the alveolar margins of the superior maxillary bones approach each other, the palate is high-arched, the lower jaw drops, and the nose and upper lip have a pinched, drawn-down look, thus causing a facial expression, considered by some due to the presence of adenoids, by others to "enlarged tonsils." I have seen marked deformity of the chest in but two cases, in both of which there were large amounts of adenoids and enormously enlarged tonsils. The facial expression, in a marked degree, is not a constant symptom, and often appears to be hereditary; and, as these growths are so often associated with hypertrophied tonsils, it can scarcely be considered more a sign of one than the other; in fact, it only becomes a symptom of adenoids, or enlarged tonsils, because they are the most usual cause of such obstruction in children. Any growth in the same region would produce the same result, unless so large that it would produce deformities of the bones by direct pressure. Such effects would only be produced by some of the solid tumors, as fibroma. These, however, are rare, and are not found in children, they occur between the fifteenth and twenty-fifth year, usually in young men. I have met but one case of true fibroma in Buffalo. This was a young man twenty-three years of age. I removed the tumor before it had produced any deformity.

The expression of the face in a case of adenoids is so different from that of atrophic rhinitis or congenital syphilis that they could not easily be mistaken. The effects of mouth-breathing upon the sleep of the child are often marked; the patient is restless, snores, wakes up frequently, and the breathing stops or intermits. After the operation is completed the difference is very marked, often so much so that the parents often fear the child is dead, on account of the contrast between the former snoring and the subsequent quiet sleep.

Obstruction of the Eustachian tubes by adenoid growths does not differ materially in its effects upon the hearing and the middle ear, from obstruction due to any other cause in the nose, naso-pharynx or tube itself, consequently a discussion of the symptomatology need not detain us. What is important, however, is the frequency with which these growths produce middle ear disease during childhood, and the marked improvement in hearing which follows their removal. Fully one-third of the cases which I have treated for adeonid growths had catarrhal or purulent middle ear disease, and as many more complained of slight deafness and tinnitus aurium, and the removal of the growths always improved the hearing, except in a case of deafness following meningitis; of course in purulent otitis, of long standing, the destructive effects of suppuration cannot be overcome; free drainage of the tube must have much to do in stopping the discharge. I shall dismiss this portion of the subject, although very important, by saying that ear complications have been most frequently the symptoms for which patients have sought relief, an unfortunate fact, for had they come under observation earlier the probability is that most of them would not have required the services of an aurist. Much of this is due to the negligence or ignorance of some family physicians, many of whom are constantly telling the parents that the child will outgrow the mouth-breathing, enlarged tonsils, etc.

The above facts show the necessity of examining the naso-pharynx of all children with middle ear disease, even when there is apparently a sufficient cause in an acute rhinitis, etc.; these may be accompanied by an increase in size of the adenoid tissue, possibly acute.

The effects on phonation of the presence of, and the subsequent removal of a tumor of, the naso-pharynx are self-evident; such a growth separates from the rest of the vocal apparatus the resonant cavity of the naso-pharynx and nose; operation restores its continuity. The bad effect of such a mechanical obstruction of phonation is, however, often increased by a paresis or laxity of the velum palate, produced by pressure of the growth against it, or from pressure of enlarged tonsils upon the descending palatine muscles, between which they are situated. Such cases have the voice peculiar to paralysis of the soft palate, and often much patience and training is required before a natural voice is regained. A small amount of adenoids producing no nasal obstruction, and no perceptible effect upon the speaking voice, will often prevent singers from giving the proper resonance and tone to the higher notes. In two cases under my care, of this nature, marked improvement was noticed in the ease with which they produced the higher notes after the growths were removed, and the fatigue following singing disappeared.

One manifestation of nasal obstruction, which is in my experience very often due to adenoids, although I have many times found it in adults with hypertrophic rhinitis, is the effect upon the patient mentally. These patients are listless, their memories are bad; in school children and college students it interferes with their progress, they are compelled to read and re-read their lessons only to forget them, and the slightest mental exertion produces a dull headache which incapacitates them for work. This condition has been named by Guye, of Amsterdam, nasal neurasthenia or aprosexia. When found in children with other symptoms of obstructed nares, open mouth, and, perhaps, some dullness of hearing, it is decidedly to their disadvantage; they appear to be naturally stupid or idiotic, and are often considered so by both parents and teachers. These children take no interest in the play of their companions, and are imposed upon by them. No one can realize the benefit that operating will bring about in these cases who has not watched the course of a few such cases.

Asthma I have not met in children with adenoids. In a young lady of twenty, asthma was apparently benefitted for a time after the removal of a spur from the septum, and adenoids; the benefit, however, was not lasting.

Jacobi has discovered certain forms of chorea minor as occurring in these cases. I have seen some peculiar facial movements produced, as far as I could judge, by the sensation of a foreign body in the throat, but I could not class these with chorea. Are such reflex symptoms not identical with habit-spasm as distinguished from chorea?

From among my cases, in which resolution had been delayed, I shall relate that of the oldest patient on whom I have operated (since writing I operated on a young lady of twenty-five for adenoids):

A countryman, twenty-four years old, consulted me in December for nasal obstruction, with severe headache, dizziness, tinnitus aurium, want of appetite and sleeplessness. On making an examination of the nose anteriorly I found marked deviation of septum to the left, and hypertrophic rhinitis; with the mirror I found posteriorly the left side of the nose filled with mucous polypi, and the vault filled with adenoid growths which obstructed the Eustachian openings and covered the upper half of the septum. Operative treatment of these conditions relieved all the symptoms in a few days, and since that time the patient has remained perfectly well. This, like most of my cases of adenoids, beyond eighteen years old, was complicated with other pathological conditions producing nasal obstruction. I do not wish to draw any conclusions from these facts. It is, however, a fact that in most of the adult patients the adenoids have been associated with obstructions, more anteriorly especially deviations of the septum.

In conclusion, I would suggest the following propositions:

1. Always suspect adenoid vegetations in children under fifteen with nasal obstruction, and do not forget their frequency in cases under twenty.

2. Defective vocal resonance, middle ear disease, and hypertrophied tonsils in children are generally due to, or associated with adenoid growths.

3. Failure to benefit middle ear disease by removing the faucial tonsils is often due to the presence of adenoids.

4. Much chronic ear trouble might be prevented by the early removal of these growths, and our percentage of deaf mutes might be perceptibly diminished by early attention to the condition of the naso-pharynx.

5. While late operation greatly improves the general health, or ear trouble, early operation would obviate many cases of both.

6. The condition of the naso-pharynx should be carefully watched after attacks of diphtheria, scarlet fever, etc.

7. Early recognition of naso-pharyngeal obstruction rests with the family physician, not with the specialist who only meets these cases after the manifestations are marked and more or less serious.

8. Physicians should not encourage the idea, although perfectly true, that the patient will outgrow this trouble, for that only occurs in many cases after much serious harm has been produced by them.

9. Should our school system ever attain an ideal perfection, it can only be when we have a board of educated medical men, as well as a Board of Regents, whose duty it shall be to examine pupils physically with special reference to the preservation of a normal condition of their eyes and ears, their chief means of obtaining ideas. The oculist would correct any errors of vision. The rhinologist or aurist would point out possible causes in the naso-pharynx of defective hearing, listlessness and defective memory, and thereby much of the apparent over-pressure of our educational institutions would be obliterated.

361 PEARL STREET.

---

THE Philadelphia County Medical Society has appointed Drs. Parvin, Hirst, Montgomery, Massey, and Baldy, a committee to conduct a series of observations relative to the effect of electricity on fibroid tumors of the uterus. It may be confidently expected that this committee will give the profession some substantial information upon this interesting subject. The Secretary of the Committee, Dr. J. M. Baldy, requests any physician who may have cases suitable, to send them to the Gynecological Out-Patient Department of the Pennsylvania Hospital, Mondays, Wednesdays, or Fridays, at 12 o'clock.

